# Italian Practice, by Antimony, Anticipated

**Published:** 1829-07-01

**Authors:** 


### Italian Method of employing Tartar-Emetic in large Doses, anticipated in this Country by Dr. Marryat.

Dr. Loudon, of Leamington Spa, has lately called our attention to the prior claims of Dr. Marryat, of Bristol, to the exhibition of large and repeated doses of tartar-emetic in inflammatory diseases, without ultimately producing any other effect on the system than that of curing the disease.

Dr. Marryat published the last edition of his Therapeutics about 1790, and died in the year 1793. The following are his words in reference to tartarized antimony in fever, which will be found to prove, that the plan adopted by Rasori, was, as nearly as possible, the same as Dr. Marryat had practised full 10 years before the time that it was first tried in Italy.

" Any fever may be soon extinguished by the use of the following powders:—Take of tartarized antimony five grains; white sugar, (or nitre) a drachm: let them be well rubbed in a glass mortar and be divided into six powders; one to be taken every three hours, notwithstanding the nausea the first may possibly occasion. If they bring on a diarrhœa, they should still be continued, and it will soon cease. If these are taken, (which is most commonly the case) without any manifest inconvenience, let there be seven grains in the next six powders; and, in the next, ten. Here I beg leave to retract what I said in some former editions of this work, viz. that till sickness and vomiting was excited, this noble medicine was not to be depended on. For I have since seen many instances wherein a paper has been given every three hours, (of which there have been ten grains in six powders) without the least sensible operation, either by sickness, stool, sweat, or urine, and though the patients had been unremittedly delirious for more than a week, with subsultus tendinum, and all the appearances of hastening death, they have perfectly recovered without any medicinal aid, a clyster every other day excepted. I have lately seen a great many cases similar to the above, and the tartarized antimony has invariably produced the same effect."

From the above passage, it will be evident that Rasori and Thommasini, and the other continental doctors, were anticipated by our countryman in the use, or, as some will say, the abuse of antimony in acute diseases—verifying the asseveration of Solomon, which need not here be repeated.—*Ed.*

